# Convalescent Plasma: A Challenging Tool to Treat COVID-19 Patients—A Lesson from the Past and New Perspectives

**DOI:** 10.1155/2020/2606058

**Published:** 2020-09-22

**Authors:** Valeria Selvi

**Affiliations:** Department of Experimental and Clinical Biomedical Sciences Radiodiagnostic Unit N. 2, University of Florence, Azienda Ospedaliero-Universitaria Careggi, Largo Brambilla 3, 50134 Florence, Italy

## Abstract

On March 11^th^, 2020, the World Health Organization declared COVID-19 infection as a pandemic. Since it is a novel virus, there are basically no proven drugs or therapies; although many laboratories in different countries are working to develop a vaccine, it will take time to make it available. Passive immunization is the therapy born from the intuition of Behring and Kisato in the late 19^th^ century. It was widely used for the treatment of bacterial infections until the discovery of antibiotics, as well as during the viral pandemics of the 20^th^ century and of the beginning of the 21^st^; it still has clinical applications (e.g., tetanus prevention). This paper summarizes the basic principles of passive immunization, with particular reference to convalescent plasma. The literature concerning its use during past epidemics and the results of the first clinical studies concerning its use during the current pandemic are discussed too. A large section is dedicated to the analysis of the possible, although rare, side effects. Recently, in 2017, the WHO Blood Regulators Network (BRN) published a position paper, recommending convalescent plasma as the first-choice treatment to be tested in the absence of authorized drugs; however, this strategy has not been followed. In the current epidemic, the principle of passive immunization through convalescent plasma has been applied in several circumstances and particularly in patients with serious complications. The first reported results are encouraging and confirm the effectiveness of plasma therapy and its safety. Also, the FDA has proposed plasma treatment in order to face the increasingly complex situation and manage patients with serious or immediately life-threatening COVID-19 disease. Several studies and clinical programs are still ongoing.

## 1. Introduction

On March 11^th^, 2020, the World Health Organization (WHO) declared COVID-19 infection as a pandemic [1].

The virus causing COVID-19 infection is a coronavirus called SARS-CoV-2; it began to scare the world since the first days of 2020 during its initial outbreak in China, because of the characteristics of contagion (high rate of contagiousness associated with high lethality) [2].

Since it is a novel virus, there are basically no proven drugs or therapies. In hospitals all over the world, there are many ongoing clinical studies. Many attempts have been made to treat seriously sick patients, using off-label drugs already known; nevertheless, to date, there is no effective targeted antiviral therapy. In most cases, drug administration has been authorized for a compassionate purpose [3]. In fact, WHO management of COVID-19 has been mainly focused on infection prevention, case detection, and monitoring; supportive care and nonspecific anti-SARS-CoV-2 treatment have been recommended [4].

Extensive vaccination is the only strategy to prevent pandemic transmission of SARS-CoV-2. Major efforts are currently being made by many laboratories in several countries to develop a vaccine; however, it will still take time before the vaccine is widely available to the population [5].

Encouraging news about passive immunization arrived from China at the end of February, and some authors reported them in their scientific publications.

Cai et al. cited two official sources (National Health and Health Commission, Health Bureau of the Logistics Support Department of the Central Military Commission; Chinese Society of Blood Transfusion) reporting significant improvements in patients affected by COVID-19 and treated with plasma donated by recovered patients [5].

Anecdotal experiences are reported by Keith et al. [6] and from Cunningham et al. [7]; in particular, the latter group reported that Biotec Group Co. announced that 10 seriously ill patients, treated with immunoglobulin therapy, demonstrated improved oxygenation and reduced inflammation and viral load [7].

In addition, Casadevall and Pirofski, referring to the news from the Xinhua news source, reported that convalescent serum was used for the therapy of 245 patients with COVID-19 in China during the first outbreak. Although few details are available and published studies involve a small number of patients, the authors concluded that convalescent serum is safe and reduces viral load [8]. Additionally, convalescent plasma could potentially be used to prevent disease in high-risk cases (vulnerable individuals with underlying medical condition, health care providers, and individuals exposed to confirmed cases of COVID-19 [8].

The literature about convalescent plasma is rapidly growing. The uniqueness of this work is that it presents all the main aspects about convalescent plasma in a single body. The already published review articles often focused on single aspects of convalescent plasma, and to the best of the author's knowledge, there are no available works covering all the main topics concerning convalescent plasma use. The present manuscript is intended to be a complete and update guide for doctors and institutions.

## 2. Methods of Research

A systematic search was conducted in major electronic databases (PubMed and MEDLINE) and Google Scholar; the applied query was “plasma OR convalescent plasma” AND “COVID-19 OR Sars-Cov-2”.

## 3. Passive Immunization: Basic Principles

Virus neutralization by antibodies is the principle behind the functioning of plasma of patients recovered from SARS-CoV-2; high-titer-specific antibodies bind to SARS-CoV-2 neutralizing the viral particles, blocking access to cells, and activating potent effector mechanisms, such as complement activation and phagocytosis [7].

There are several ways to achieve passive immunization.

Antibodies can be delivered to the recipient by (i) human whole blood, (ii) human or animal plasma or serum, (iii) pooled human immunoglobulin for intravenous (IVIG) or intramuscular (IG) use, (iv) high-titer human immunoglobulin for intravenous or intramuscular use from immunized or convalescing donors, and (v) monoclonal antibodies (MAb) [9, 10]. Transfusion of whole blood to provide convalescent plasma should be avoided unless its use is clinically indicated; collection of convalescent plasma should be performed only by apheresis to avoid unnecessary red cell loss in the donor [11]. Plasma administration is the preferred method to provide passive immunity in pandemic scenarios at least in the immediate term; usually, immunoglobulins are prepared by fractionating large pools of human plasma collected from approximately 10,000–40,000 donors [12]. Moreover, plasma administration represents a rapid and effective therapy, has lower costs than other methods [13], and presents a broader spectrum response. Studies suggest that it not only neutralizes the pathogen but also provides passive immunomodulatory mediators allowing the recipient to control the excessive inflammatory cascade induced by the infectious agent [14]. Animal plasma collection should be avoided if possible as it can cause side effects collectively called “serum sickness” [15].

## 4. Convalescent Plasma: A Lesson from Past and Current Applications

Convalescent plasma is not a novel therapy; it is a therapy widely used in the past, both for bacterial and viral pathologies.

Behering and Kisato were the first in 1890 to provide the basis of passive immunization, then known as serum therapy. Despite limited knowledge on the structural and functional complexity of antibodies, they demonstrated that not previously immunized animals can be protected from sublethal doses of diphtheria and tetanus toxin with serum therapy. The discovery was so important that in 1901, Behring earned his Noble Prize for it. Given the early success in the 1900s, passive immunization was rapidly expanded; it was used to treat several bacterial infections including Corynebacterium diphtheriae, Streptococcus pneumoniae, Streptococcus pyogenes, Clostridium tetani, Haemophilus influenzae, and Neisseria meningitidis; type-specific antipneumococcal serum was used as the first-line treatment for lobar pneumonia [15]. During the first half of the 20^th^ century, serum therapies were successfully used to treat patients affected by many infectious diseases (anthrax, plague, scarlet fever, measles, tularemia, diphtheria, dysentery, meningococcal meningitis, rabies, and pneumococcal pneumonia) [16].

However, serum therapy for bacterial diseases suddenly stopped after the discovery of antibiotics. Moreover, the tools for the correct selection of plasma were still missing; the risk of serum disease was very high in those years, as plasma was frequently prepared from the blood of hyperimmunized animals [15].

Regarding viral diseases, studies about convalescent plasma use to fight pandemics of the last century are available; the reported results were positive [8].

In the early 20^th^ century, convalescent serum was used to fight outbreaks of viral diseases such as poliomyelitis, measles, mumps, and Spanish influenza [8].

A meta-analysis by Luke and colleagues reported eight studies involving 1,703 patients with 1918 influenza pneumonia from 1918 to 1925. Patients were often selected among the most serious ones and received an infusion of influenza convalescent human blood products; the outcome of the treated patients was compared to that of the untreated influenza pneumonia controls. The study showed a pooled absolute reduction of 21% in the mortality rate compared to controls. Unfortunately, the included studies were few and with methodologic limitations (no study was a blinded, randomized, or placebo-controlled trial; moreover, convalescent sera were developed and used in many cases without measuring antibody titers or without knowledge about viral serotypes); anyway, this treatment received consensus at the time, and it was applied in several countries [4, 8, 17].

In the modern era, the treatment of Argentine hemorrhagic fever (Junin virus) with convalescent immune plasma was applied as part of a nationally organized response; patients treated with immune plasma had a much lower mortality than those given normal plasma [16, 18].

Convalescent plasma or immunoglobulins were administered as a last chance to reduce the mortality rate of patients with SARS; several studies showed a shorter hospital stay and lower mortality in patients treated with convalescent plasma compared to those not treated with convalescent plasma. The largest study involved the treatment of 80 patients showing clinical deterioration despite treatment with methylprednisolone. Earlier plasma administration was more likely to be effective: patients treated before the 14^th^ day had better prognosis compared to those treated later. In addition, patients who were PCR positive and seronegative for coronavirus at the time of therapy had improved prognosis. No immediate adverse reactions were observed [4, 8, 19].

Positive evidence was reported for the treatment of influenza A (H5N1) too [20].

Regarding the pandemic 2009 influenza A H1N1, the results from the prospective cohort study by Hung and colleagues showed that plasma treatment reduced mortality (the patients involved in the study were seriously ill and required intensive care); no adverse events were observed [4, 8, 20]. A second trial by Hung and colleagues was conducted on 35 patients affected by severe influenza A H1N1 during 2010 and 2011, using immunoglobulins fractionated from plasma of patients recovered from the previous 2009 influenza A H1N1. Treated patients showed a lower viral load and reduced mortality rate within 5 days of symptom onset [4, 8, 21].

A meta-analysis by Mair-Jenkins and colleagues, including 32 studies of SARS coronavirus and severe influenza, reported that convalescent plasma reduced mortality and it was safe (no relevant adverse events or complications after treatment were reported). The reduction of mortality was higher when convalescent plasma was administered earlier after symptom onset [22].

Regarding Ebola disease, the use of convalescent plasma was recommended by the WHO in 2014 as an empirical treatment during the outbreaks [8]. The first use of convalescent plasma for Ebola dates back to previous times. The study of Mupapa et al. involved eight patients during an outbreak in 1995; among these, seven survived [23]. The Ebola-Tx clinical trial tested the efficacy of convalescent plasma as a treatment for Ebola in Guinea: the trial confirmed convalescent plasma safety, but unfortunately, the efficacy was not proven. No association with the dose of neutralizing antibodies was apparently found, even if the levels of neutralizing antibodies were low in many plasma donations. The authors concluded that further studies were needed to assess the effectiveness of antibody doses higher than those used in their study [24]. Sahr et al. used convalescent serum in Sierra Leone for Ebola treatment: their study revealed a significantly lower fatality rate for patients treated with convalescent whole blood with respect to those receiving standard treatments [13].

A protocol for convalescent plasma in the treatment of Middle East Respiratory Syndrome (MERS) caused by a coronavirus was established in 2015. Three patients with MERS in South Korea were treated with convalescent serum, but only two showed neutralizing activity. The authors concluded that high antibody titer (≥1 : 80) should be needed to achieve good neutralization activity [4, 8, 25].

Keller and Stiehm listed all the pathologies for which passive immunization has been or is currently being used. For each pathology, they specified when passive immunization is to be used for prevention versus treatment and if the efficacy has been demonstrated (they also pointed out when there is no recommendation to use the passive immunization tool, even in the case of demonstrated efficacy). More than 30 infectious pathologies were analysed. The efficacy of passive immunization in the prevention of infectious diseases has been proven for tetanus, Clostridium botulinum, hepatitis A, hepatitis B, RSV (respiratory syncytial virus), CMV (cytomegalovirus), VZV (varicella zoster virus), rabies, measles, and vaccinia. In addition, passive immunization has been proven but not recommended for the treatment of respiratory infections (Streptococcus, Streptococcus pneumoniae, Neisseria meningitidis, and Haemophilus influenzae) or for enterovirus infection. The efficacy of passive immunization in the treatment of infectious disease has been proven for diphtheria, tetanus, Clostridium botulinum, and vaccinia and has been proven but not recommended for respiratory infections (Streptococcus, Streptococcus pneumoniae, Neisseria meningitidis, and Haemophilus influenzae), parvovirus, and enterovirus [9].

## 5. Safeness of Convalescent Plasma: Exploring the Rare Side Effects of Plasma Therapy

As already discussed, previous studies on convalescent plasma in pandemic scenarios have shown that plasma is a safe treatment [4, 8, 13, 17–25].

Anyway, side effects are possible for any medication; MacLennan and Barbara analysed the possible side effects of generic plasma administration (not only convalescent plasma) in a recipient. Several factors can lead to adverse events (donor-related factors, which testing is performed on plasma, any treatment or modification to which it has been subjected, interaction between donor factors, and the patient's immune system).

Possible adverse reactions can be classified into three groups: immune reactions (anaphylactic/anaphylactoid reactions, mild allergic reactions, haemolysis, and transfusion-related acute lung injury), physicochemical reactions (fluid overload, citrate toxicity, and chemicals), and infectious risks.

Anaphylactic reactions are uncommon, but severe and potentially life-threatening (in 2003, the incidence in the UK was approximately 0.002%). IgE mediates anaphylactic reactions; the term “anaphylactoid” describes a similar reaction not mediated by IgE.

Less severe allergic reactions are much more common and usually characterized by cutaneous symptoms, ranging from mild pruritus to urticaria and flushing.

Haemolysis can occur following transfusion of plasma containing high-titer anti-A or anti-B haemolysins to an A or B recipient. It could be very serious, and deaths have also been reported. To avoid this reaction, plasma should always be ABO compatible; if not possible, the plasma should be tested for haemolysins and found negative for high titer of them. Concomitant transfer of antibodies against other red cell antigens might occasionally cause haemolysis in the recipient. Thus, donor screening procedures to detect clinically significant antibodies are essential to minimize this risk [26].

TRALI is an acute respiratory reaction, indistinguishable from the adult respiratory distress syndrome (ARDS), occurring in association with transfusion of blood components; the incidence was reported variously, ranging from 1 in 5,000 to 1 in 50,000. It is caused by the presence of antibodies against leucocyte antigens (HLA antigens seem the most frequent [11]) in donor plasma [26]. To avoid this risk, preference should be given to the use of plasma from male donors or from females who have never been pregnant, including abortions. This measure lowers the possibility to find antibodies against HLA or granulocyte antigens causing TRALI in the donor plasma [11].

Moreover, it was reported that the presence of certain antibodies may cause immune enhancement of pathogenicity, termed ADE (antibody-dependent enhancement), for several viral diseases, such as dengue virus and SARS [27].

Physiochemical reactions can sometimes be severe, but usually not life-threatening:

- Fluid overload is one of the most common complications of transfusion and can lead to pulmonary oedema
- Citrate toxicity depends on the action of citrate in binding calcium and therefore in reducing the availability of ionised calcium for normal neuromuscular function; it is not frequent because citrate is rapidly metabolised by the liver
- Some units of plasma might contain chemicals (e.g., drugs) derived from the donor to which the recipient might react

Modern technologies allow to minimize infectious risk. Firstly, bacterial transmission is not a significant risk factor as the plasma is frozen within hours of collection and processing. Secondly, an accurate selection of donors and pathogen reduction processes can be applied to minimize viral infection risk (for single-unit components, methylene blue is used; for plasma pools, solvent detergent is used) [26].

Specific side effects are identified for single plasma components; immunoglobulins have been associated with thrombotic events, renal toxicity, and aseptic meningitis [26]. Tamburello and Marando reported that treatment with human immunoglobulin during the SARS-CoV-2 pandemic was associated with a significantly increased risk of same-day thrombotic events (from 0.04 to 14.9%) [3]. However, the estimated risk of serious adverse events is less than 5% [26].

A recent work by Joyner and colleagues explores the safeness of the use of convalescent plasma in 20,000 critically ill COVID-19 patients. The cohort studied is very huge; thus, the reported results should be considered particularly reliable. Serious adverse events within 4 hours of completion of COVID-19 plasma transfusion were 146 (less than 1% of all transfusions). 50 events were judged surely unrelated to plasma transfusion. Among the other events, there were 83 nonmortality events reported (37 reports of transfusion-associated circulatory overload, 20 reports of transfusion acute lung injury, and 26 reports of severe allergic transfusion reaction). 13 mortality events happened; they were judged only as possibly, not definitely, related to the transfusion of COVID-19 convalescent plasma. Notably, the vast majority of other serious adverse events, which happened within seven days of completion of the convalescent plasma transfusion, were judged to be unrelated to the plasma transfusion [28].

## 6. Convalescent Plasma: Recommended Therapy in the Context of a Pandemic

Recently, in 2017, the WHO Blood Regulators Network (BRN) published a position paper, recommending the need for healthcare systems to prepare adequate infrastructures to deal with the emergence of any pandemic caused by new emerging viruses; in that paper, the BRN suggested plasma from recovered patients as the first-choice treatment to be tested. Based on the evidence from past experience in passive immunization, the BRN explained that there was a considerable possibility that the application of whole blood (as well as plasma, serum, or immunoglobulin concentrates) from convalescent persons could be effective in the treatment/prevention of infectious disease. Thus, in the absence of effective vaccines and antiviral therapies for the emerging pathogen, an organized program to collect convalescent plasma or serum from disease survivors could provide a potentially valuable empirical intervention, while data on the effectiveness and safety of its use are obtained through orderly scientific studies [16]. In fact, any blood derivative should be considered a drug, and if administered for different indications from the authorized ones, it must be tested for the specific new application [29].

Epstein and Burnouf updated the BRN recommendations to the current pandemic caused by SARS-CoV-2 [11].

## 7. Results of the First Studies regarding Convalescent Plasma for the Treatment of COVID-19

Several case reports and case series reporting convalescent plasma for COVID-19 patient treatment are available; most of them show positive results in terms of efficacy and safety of the convalescent plasma for treating COVID-19 [30]. The first case series was published by Shen et al. at the end of March 2020. The authors reported a case series of 5 critically ill patients with acute respiratory distress syndrome (ARDS) under mechanical ventilation. The study compared the clinical outcomes before and after the transfusion. It was observed that ARDS resolved in 4 patients at 12 days after transfusion; in addition, 3 patients were discharged, and 2 patients were in stable conditions [31].

Unfortunately, data from case reports and case series are observational, and they are not sufficient for a definitive validation of the treatment.

Some controlled trials are already available too; they confirm the outcomes of the first case series.

A randomized control trial out of Wuhan was the first to be published: 103 patients with severe or life-threatening COVID-19 (52 in the convalescent plasma-treating group and 51 in the control group) were enrolled, but unfortunately, the study had an early termination due to low patient enrollment as the regional outbreak waned. Contrary to expectations, the study failed to detect a statistically significant difference in the evaluated outcomes (time to clinical improvement, 28-day mortality, and time from randomization to discharge). However, convalescent plasma was demonstrated to be associated with antiviral activity in patients with COVID-19 (convalescent plasma treatment was associated with higher rates of negative SARS-CoV-2 viral PCR results from nasopharyngeal swabs at 24, 48, and 72 hours); a statistically significant improvement was noted for the convalescent plasma treatment group compared to controls in the subgroup of patients without life-threatening COVID-19 (91% improvement in the plasma group compared to 68% in the control arm). The median between the onset of symptoms and the beginning of the treatment was 30 days. This time window could have affected the study to detect a clinically important benefit of the convalescent plasma therapy, in addition to the early termination of the study and to the type of patient conditions (only severe or life-threatening disease) [32].

The researches of Hartman et al., of Liu et al., and of Salazar et al. confirmed that early convalescent plasma administration is of greater clinical benefit than delaying transfusion in patients with severe disease.

Hartman et al. described a series of 31 patients (16 patients with severe disease and 15 patients with life-threatening disease). They demonstrated that convalescent plasma is associated with reducing ventilatory requirements in patients with both severe and life-threatening diseases [33].

These results are consistent also with a recent cohort study by Liu and colleagues. In this study, 39 patients were treated with convalescent plasma and were compared to 156 control patients. The authors reported a lower mortality rate among patients with severe or worse disease who received convalescent plasma and significantly better outcomes among patients transfused prior to mechanical ventilation [34].

Salazar et al. enrolled 387 patients (136 transfused patients and 251 nontransfused control COVID-19 patients); they found that patients transfused within 72 h of hospital admission had decreased mortality within 28 days, whereas patients transfused after 72 h of hospital admission did not. These data demonstrate that early convalescent plasma transfusion after hospital admission reduces mortality within 28 days posttransfusion [35].

Two other articles deserve to be mentioned, the one of Duan et al. and the other of Joyner et al.

Duan et al. compared 10 severely ill patients treated with convalescent plasma to a historical control group of 10 severely ill patients not treated with convalescent plasma. The COVID-19-transfused patients' group showed better clinical outcomes than the historical control group. All enrolled severe COVID-19 patients had improvement of clinical symptoms and showed different degrees of absorption of the pulmonary lesions after convalescent plasma transfusion. The authors showed amelioration of routine laboratory criteria and pulmonary function (lymphocytopenia, an important index for prognosis in COVID-19, tended to be improved after convalescent plasma transfusion). Increase of neutralizing antibody titers was demonstrated [36].

Joyner et al. studied the effects of convalescent plasma use in a very huge cohort of 20,000 critically ill hospitalized patients. The aim of the paper differs from the ones previously mentioned: the study was designed to demonstrate the safety of convalescent plasma. Anyway, the seven-day mortality rate in this extremely high-risk cohort of patients was 8.6% only. The authors anticipated the intent to create a control comparator group using patients hospitalized with COVID-19 during the same period; they will discuss potential convalescent plasma efficacy in a future publication. Given the large number of observations, it is expected that the results of this study will have significant importance in evaluating the efficacy of the treatment and its reliability [28].

Moreover, there are several ongoing randomized controlled trials on the role of convalescent plasma to treat COVID-19 (Zheng et al. estimated that the main underway trials are 48 in the world). Also in this case, a positive confirmation of the results in terms of the efficacy of the treatment for COVID-19 is strongly expected [37].

## 8. Convalescent Plasma for COVID-19: Practical Points

Preparation requirements for convalescent plasma follow the standard operating procedures for plasma collection and all applicable regulations. Thus, health system requirements are the same for routine plasma collection procedures via plasmapheresis. During plasma donation procedure, the blood cells and plasma are removed from the body and separated by a plasmapheresis machine; then, the blood cells are returned to the donor while plasma is collected. Plasma products are stored as fresh-frozen plasma, until usage. Recently, approved serological assays are necessary to detect SARS-CoV-2 (RT-PCR test) in serum and virologic assays [35].

Regarding plasma treatment in the context of the current pandemic, the following points are worth remarking.

Plasma should only be collected from selected recovered individuals diagnosed with COVID-19 for at least 3 weeks. At least 14 days must have elapsed since complete recovery, in order to minimize the possible risk of SARS-CoV-2 in the blood. The titer of anti-SARS-CoV-2 IgG should be determined, and virus inactivation procedures should be strictly attended before using plasma [5]. Actually, the recommended viral neutralization titer cut-off for COVID-19 convalescent plasma is at least ≥1 : 160. This corresponds to a receptor binding domain IgG titer ≥ 1 : 1350 [35]. A titer of 1 : 80 may be considered acceptable if an alternative matched unit is not available [38]. Although largely experimental, the optimal dose of convalescent plasma to be administered to a COVID-19 patient ranges between 200 and 500 ml [28, 31–33, 35, 36].

Treatment effectiveness is expected to be better when immune plasma is collected from patients of the same city, or surrounding area, since it is assumed that these donors have defeated the same virus (virus genome can mutate); likewise, lifestyle, diet, and environment play an important role in the development of specific antibodies against the virus [39].

As a principle, convalescent plasma should be used as soon as possible in the acute stage of the disease of the recipient [4, 5], and it is important that the titer of anti-SARS-CoV-2 antibodies be high.

It is essential to ensure ABO compatibility between donor and recipient; theoretically, transfusion of plasma from at least two donors may be better to achieve more effective immune protection from delivery of diverse antibodies. Standard selection criteria for plasma donation, according to local requirements, must always be followed, as well as standard postdonation treatment of plasma [11].

## 9. Perspectives

Unfortunately, the BRN recommendations have been disregarded. At the beginning of the present pandemic, no healthcare system had already organized programs to collect convalescent plasma or serum from recovered patients to fight a potential new emerging viral pathogen.

Currently, some clinical studies and programs have started, but unfortunately, the procedures are very slow [7, 40, 41]. The first reported results are very encouraging and confirm the effectiveness of plasma therapy and its safety [42–44].

The classical process to approve a new drug for clinical use is long, but a terrible pandemic emergency is underway and the time to wrest the fate of many people from death is very short.

It is essential that governments follow the strategy recommended by the BRN in 2017. Recently, the Food and Drug Administration (FDA) has also moved on this path, in consideration of the numerous evidences of efficacy and safety of plasma therapy coming from past experiences and the first scientific confirmations in the current pandemic. The administration has remarked the importance to study the safety and efficacy of COVID-19 convalescent plasma enrolling patients in clinical trials. In addition, two other strategies have been authorized to allow patients to access treatment. Firstly, it provides an expanded access for the use of COVID-19 convalescent plasma dedicated to patients with serious or immediately life-threatening COVID-19 disease, who are not eligible or unable to participate in randomized clinical trials (21 CFR 312.305). Secondly, given the public health emergency, FDA facilitates access to COVID-19 convalescent plasma for patients with serious or immediately life-threatening COVID-19 infections: the patient's physician can request a single emergency Investigational New Drug Application to obtain expanded access for an individual patient (21 CFR 312.310) [29]. This strategy could allow to save as many lives as possible.

In addition, the FDA is promoting an awareness campaign to invite patients recovered from COVID-19 to donate plasma [45].

Expected results from ongoing trials should definitely support the researches in finding the best criteria for including/excluding convalescent plasma in COVID-19 patients' treatment. As detailed in the available studies, performed analysis suggests convalescent plasma for the most serious cases and at their early stage. Thus, the early recognition of the COVID-19 patients who may develop critical illness is the key question for convalescent plasma treatment. They are the patients to be treated with convalescent plasma. It is known that most mild COVID-19 patients can be self-recovered, and convalescent plasma may be inappropriate therapy for them. And for end-stage COVID-19 patients, the convalescent plasma treatment may not be able to regress the poor outcome as demonstrated by the current studies [46].

## 10. Conclusions

The strategy of the FDA seems the most correct, since convalescent plasma appears to be a safe and effective therapy, and a vaccine requires a long time to get ready. To date, there are no other authorized therapies against SARS-CoV-2. Furthermore, it should not be forgotten that the other currently applied therapies (e.g., antiviral drugs and hydroxychloroquine) have remarkable side effects and are administered for compassionate use [7, 47].

Another key point is that convalescent plasma should be hyperimmune and contain high antibody titers against SARS-CoV-2. It is still unknown how long patients have good antibody levels in their blood [48]; therefore, at least hypothetically, the time window for convalescent plasma donation is limited to the first period after a patient's full recovery. Fortunately, many patients in the world are recovering from COVID-19 infection.

This should be the right time to donate plasma to treat seriously ill patients. Governments should be aware of this opportunity and start organizing appropriate plasma donation campaigns and adequate plasma collection programs.
